# Acute and Subchronic Oral Toxicity Study of Gardenia Yellow E500 in Sprague-Dawley Rats

**DOI:** 10.3390/ijerph17020531

**Published:** 2020-01-14

**Authors:** Xiaoqiao Tang, Yangfeng Wang, Wenxiang Yang, Yanhua Zheng, Chunxia Liu, Min Qu, Haibin Xu, Lei Zhang, Jiang Liang, Bolin Fan

**Affiliations:** 1 Hubei Provincial Key Laboratory for Applied Toxicology, Hubei Provincial Center for Disease Control and Prevention, Wuhan 430079, China; melon_qiao@163.com (X.T.); wyfviolet@163.com (Y.W.); ywx_21@163.com (W.Y.); zyh512995587@163.com (Y.Z.); lchx216@163.com (C.L.); minqu2977@163.com (M.Q.); 2 Fuling District Center for Disease Control and Prevention, Chongqing 408000, China; 3 China National Center for Food Safety Risk Assessment, Beijing 100022, China; hbxu1231602@cfsa.net.cn (H.X.); zhanglei@cfsa.net.cn (L.Z.)

**Keywords:** gardenia yellow, colorant, *Gardenia jasminoides*, subchronic toxicity, no observed adverse effect level

## Abstract

Objective: This study was conducted to evaluate the acute and subchronic toxicity of gardenia yellow, a natural colorant widely used in China and other Asian countries. An acute toxicity test was performed in S-D rats of both genders and the lethal dose (LD_50_) of per oral gardenia yellow was estimated to be more than 15.0 g/kg·bw. In the subchronic study, gardenia yellow was orally administered to rats by gavage at doses of 0, 0.50, 1.50 and 4.50 g/kg·bw/day for 90 days followed by a recovery period of 28 days. No appreciable toxic-related changes were observed in the 0.50 g/kg·bw/day group. When the animals received gardenia yellow at 1.50 g/kg·bw/day or more, body weight loss was observed, and pigments began to deposit in several vital organs, resulting in significant changes of several hematological and biochemical indicators related to the nutritional status of the body, liver and kidney function, more severe in the high dose group. In the recovery period, the alterations of the clinical symptoms and parameters were relieved a lot. Based on the results of the current study, the no observed adverse effect level (NOAEL) of gardenia yellow E500 in rats was set to be 0.50 g/kg·bw/day.

## 1. Introduction

Gardenia yellow is the fruit extracts from *Gardenia jasminoides Ellis*, an evergreen shrub probably originated from China, which has been popularly used as a traditional Chinese herb for a long history [1,2,3]. For the characters of good stability and strong coloring power, gardenia yellow is now widely used as a natural colorant for foods in East Asia, especially in China, Japan, South Korea and Taiwan [2,4,5]. In the Chinese National food safety standard for the use of food additives [6], gardenia yellow is approved to be used in 22 varieties of food products, including fat emulsions, vegetables, crackers, confectionery, coffee, tea, snacks, etc.

The products of gardenia yellow usually consist of over 20 species of crocetin esters with the amount of more than 95% (*W*/*W*). Among them, the majority, usually 45%~55% (*W*/*W*), is crocin. The other 5% (*W*/*W*) components are iridoid glycosides with 0.2%~1% (*W*/*W*) geniposide [7,8]. Gardenia yellow has raised a safety concern for the toxicity of its main components published in the previous studies. The study of Li et al. showed that crocin was of low acute toxicity with the LD_50_ in the range of 1–5 g/kg·bw, varying for different purities and different animal species [9]. While the 90-day orally administration of crocin to rats was reported to result in the early damage to hepatocyte of rats with increased aminotransferase (AST) levels at the doses of l43~166 mg/kg·bw [9]. Moreover, as the important bioactive component in *Gardenia jasminoides*, geniposide is considered to be the main toxic substance in gardenia yellow [10,11,12]. Geniposide is considered to be hepatotoxic in rats after conversion into genipin [10]. In the study of Ding et al., geniposide was reported to lead to increased liver enzymes, increased liver weight and hepatic necrosis in S-D rats at a single dose of 574 mg/kg or more [13], while the present studies noted that long-term administration of geniposide at the pharmacodynamic doses (72.9 mg/kg·bw/day) would not cause liver damage in rats [13]. In addition, geniposide-induced nephrotoxicity was also reported in rats following 90-day oral administration of 100 mg/kg·bw/day [14]. Thus, in the current China National Food Safety Standards (GB7912-2010) [15], gardenia yellow product is required with the maximal limits of 1% (*w*/*w*) for geniposide. However, the existing toxicological studies focused on its components are still insufficient to assess the safety profiles of gardenia yellow as a whole product, and the chromatic valences of the tested gardenia yellow were not mentioned. Moreover, as an early approved food additive, the toxicity of gardenia yellow product has not been systematic assessed according to the international testing guidelines and regulatory requirements. With the increasing production and demand of gardenia yellow in recent years, it is necessary to reassess the safety of gardenia yellow. Thus in the current research, in compliance with the Organization for Economic Co-operation and Development (OECD) guidelines for the Testing of Chemicals [16] and Chinese Good Laboratory Practice (GLP) Standard, the comprehensive acute and subchronic toxicological studies of gardenia yellow E500 using S-D rats were undertaken and the NOAEL was determined, which will provide a basis for its risk assessment.

## 2. Materials and Methods

### 2.1. Preparation and Characterization of the Test Substance

Gardenia yellow sample E500 (Lot. 20150529) was provided by Henan Zhongda Hengyuan Biotech Co., Ltd., of which chromatic valences were 510. The dried ripe fruits of *Gardenia jasminoides*, collected from Hubei province, were ground into fine pieces and extracted with 80% (*v*/*v*) alcohol (food grade, Lot No. 20141223, from Henan Xinheyang alcohol Co., LTD), then concentrated by macro-porous resins and further dried by a spray drying to form final product, colorant powders. No toxic chemicals were involved in manufacturing. The amounts and composition of crocetin and its derivative of gardenia yellow were determined by the HPLC-ACPI-MS-MS method which has claimed the patents both in China (No. 201610124837.6) and U.S. (U.S. 2017/0248561 A1).

In the following tests, gardenia yellow powders were dissolved to indicated dosing concentrations by 0.5% sodium carboxymethyl cellulose solution. These solutions were stored in white plastic bottles at 4 °C and prepared twice a week.

### 2.2. Experimental Animals

The studies were conducted at the Hubei Provincial Academy of Preventive Medicine, the GLP-certified laboratory (GLP16002082). Males and females of Specific-pathogen-free (SPF) Sprague-Dawley (S-D) rats were purchased from Hubei Experimental Animal Research Center (license number: SCXK(Hubei)2015-0018). Every two animals of the same sex were housed in an IVC cage (40 air exchanges/hr). All the cages were placed in a controlled illumination room (10 h light/14 h dark cycle) with controlled temperature (20–25 °C) and humidity (40%–70%). Diet (standard rodent diet) and tap water were given ad libitum.

### 2.3. Acute Oral Toxicity Study

An acute toxicity test was performed in 20 healthy S-D rats of both genders with the body weight ranging from 180 to 220 g. After being fasted overnight, all the rats were gavaged 3 times within 24 h with 20 mL/kg·bw for each dose. Since the maximum suspension concentration of gardenia yellow powder in 0.5% sodium carboxymethyl cellulose solution was measured as 0.33 g/mL, the final dose of gardenia yellow to rats reached 15.0 g/kg·bw. On the day of administration, the rats were observed closely for about half an hour after each gavage and once daily until the end of the observation period of 14 days. Mortality and clinical signs were monitored every day and the body weight was recorded once weekly. At the end of the experiment, all the rats survived were anesthetized by carbon dioxide and then sacrificed for a grossly anatomy.

### 2.4. Subchronic Oral Toxicity Study

#### 2.4.1. Animal Assignment and Treatment

The 90 days subchronic toxicity test was performed in 120 healthy S-D rats of both genders with an average body weight ranging from 60 to 80 g. After 5 days of acclimatization, they were randomly assigned into four groups, i.e., a vehicle control group and three treatment groups. The main groups with 10 rats/sex/group received doses of 0.00, 0.50, 1.50, 4.50 g/kg·bw/day by gavage for 90 days and were sacrificed thereafter. Meanwhile, satellite groups, or the parallel control group and the high-dose group with 10 rats/sex/group were treated with gardenia yellow in the same way for 90 days then followed by a recovery period of 28 days. Based on the maximum tolerable intragastrical concentration of gardenia yellow for repeatedly dosing as 0.3 g/mL and the appropriate intragastric volume for repeatedly dosing as 15 mL/kg·bw, the high dose was set to be 4.50 g/kg·bw/day. A low dose of 0.50 g/kg·bw/day and a medium dose of 1.50 g/kg·bw/day were determined by a common ratio of 3.

All the experimental procedures were examined and approved by the Animal Management and Use Committee of Hubei Food and Drug Safety Evaluation Center (approval No.: 201602014).

#### 2.4.2. General Clinical Observations

Animals were monitored daily for abnormalities and mortality throughout the experiment. Eye examination (cornea, conjunctiva, iris, crystalline lens) was performed on rats in the high-dose group and the control group before and at the end of the experiment. Body weights were recorded twice a week in the first month and once a week thereafter. Food consumption data were collected once weekly.

#### 2.4.3. Clinical Investigations

Urinalysis was performed approximately one week before the scheduled sacrifice. Urine samples from all of the surviving rats were collected in stainless-steel metabolism cages for approximately 20 h. The following parameters were recorded with a urine analyzer (Urist-100a, China): pH, glucose (GLU), protein (PRO), occult blood (BLD), specific gravity (SG), bilirubin (BIL), urobilinogen (URO), ketone body (KET) and white blood cell (WBC).

At the end of the treatment or recovery period, rats were fasted overnight and anesthetized with pentobarbital sodium. Blood samples for hematology analysis were collected from tail vein and those for coagulation function detection and biochemistry analysis were collected from the abdominal aorta. Hematology was measured using an automatic blood analyzer (Sysmex XT-2000iv, Japan). The following parameters were analyzed: white blood cell (WBC), red blood cell (RBC), hemoglobin (HGB), hematocrit (HCT), platelet (PLT), mean corpuscular hemoglobin (MCH), mean corpuscular hemoglobin concentration (MCHC), neutrophil (NEUT%), percentages of lymphocyte (LYMPH%), monocyte (MONO%), eosinophil (EO%) and basophil (BASO%) and reticulocyte (RET%). Activated partial thromboplastin time (APTT) and prothrombin time (PT) were measured by a coagulation function analyzer (Sysmex CA-510, Japan). Clinical biochemistry parameters, such as alanine aminotransferase (ALT), aspartate aminotransferase (AST), total protein (TP), albumin (ALB), alkaline phosphatase (ALP), glucose (GLU), blood urea nitrogen (BUN), creatinine (CREA), cholesterol (CHOL) and triglyceride (TG), were analyzed by an automatic biochemical analyzer (Backman AU-680, America). Total bilirubin (TBIL) was measured by the method of enzyme-linked immunosorbent assay because the absorbance of gardenia yellow was similar to that of TBIL.

#### 2.4.4. Pathology Examinations

A complete gross necropsy was performed and the selected organs (i.e., brain, thymus, heart, livers, spleen, kidneys, adrenal gland, uterus, ovaries, testes and epididymides) were isolated and weighted. Relative organ weight was expressed as the ratio of organ to the brain. The following organs and tissues were fixed and preserved in 10% neutral buffered formalin: brain (representative regions including cerebrum, cerebellum and medulla/pons), pituitary, eyes, spinal cord (cervical, thoracic, lumbar), thymus, trachea, oesophagus, thyroid, parathyroid, salivary gland, heart, aorta, lung, liver, spleen, kidneys, adrenals, stomach, duodenum, pancreas, jejunum, ileum, colon, rectum, lymphonodi mesosteniales, testes, epididymides, uterus, ovaries, urinary bladder, prostate, breast, skin, muscles and sternum. All organs and tissues from the control and high-dose group and lungs, trachea, kidney, thyroid gland and parathyroid gland from the lower dose groups were embedded in paraffin and sectioned. After being stained with hematoxylin-eosin, the slides were examined with a microscope (Thermo CX41F, America) to detect any lesions.

### 2.5. Statistical Analysis

Response variable values were analyzed for each gender and presented as the mean ± standard deviations. Numerical data such as body weight, food consumption and hematology parameters were analyzed using a one-way analysis of variance (ANOVA) test and multiple comparison of Dunnett’s t-test when the variance data were homogeneous. Count data, such as urine parameters, were measured using the Kruskal–Wallis rank sum test. Statistical significance was defined as the *p*-values less than 0.05 (*p* < 0.05).

## 3. Results

### 3.1. Characterization of Gardenia Yellow Sample

**Figure 1:**
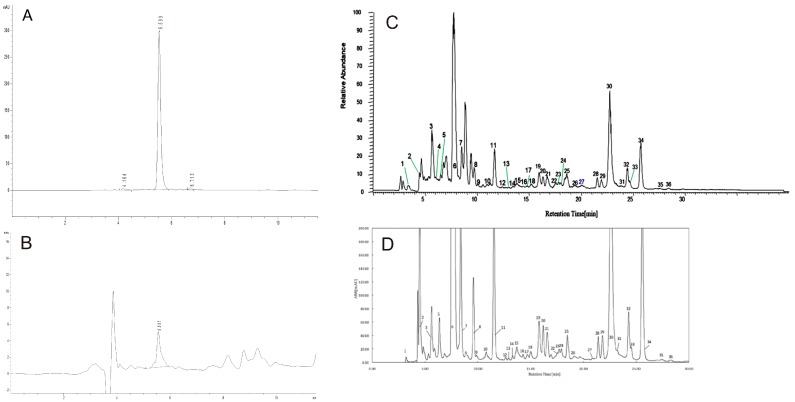
HPLC and UPLC-MS/MS chromatogram of the crocin and geniposide from gardenia yellow (E500) (**A**) HPLC Chromatogram of Standard Genipin (water as solvent), (**B**) HPLC Chromatogram of Gardenia yellow sample (E500), (**C**) HPLC chromatogram of crocetin derivatives, (**D**) Total ion chromatogram of crocetin derivatives from gardenia yellow (E500).

The final gardenia yellow product is an orange-red colored powder. Approximately, 50 kg of dried fruits can produce 1 kg of gardenia yellow (E500). As shown in Figure 1, the gardenia yellow samples were analyzed with 36 fractions of crocetin and derivative, which accounted for 44.82% (*W*/*W*) of the total. The geniposide content of the sample, analyzed by HPLC Chromatogram, was calculated to be 0.234% (*W*/*W*).

### 3.2. Oral Acute Toxicity Study in Rats

Except one accidental and treatment-irrelevant death on the 7th day after treatment, the surviving rats treated with gardenia yellow at the dose of 15.0 g/kg·bw were observed with no obvious abnormality during the 14-day observation period. On the first day after administration, the treated rats were observed with dark red stool and the orange-red urine, which were back to normal the next day. The body weight gain and gross examination at autopsy revealed no treatment related changes in both genders (data not shown). Thus, the LD_50_ of gardenia yellow to S-D rats was estimated to be more than 15.0 g/kg·bw.

### 3.3. Subchronic Toxicity Study in Rats

#### 3.3.1. General Condition, Body Weight and Food Consumption

During the experimental period, there was no mortality in groups treated with gardenia yellow of 0, 0.5 and 1.5 g/kg·bw/day. No notable signs were found in general conditions of the low dose groups, as compared with the control group. Yellowish skin, orange-colored urine, loose stools and diarrhea were observed in most animals from medium and high dose groups, accompanied by the decreasing tendency of body weight gain and food intake, which were considered dose-related. At the end of the treatment, there were significantly decreased mean body weight in the females of the medium-dose group, and significantly decreased mean body weight and food intake in the high-dose group of both genders, with 2 males and 4 females dead before the scheduled necropsy.

**Figure 2:**
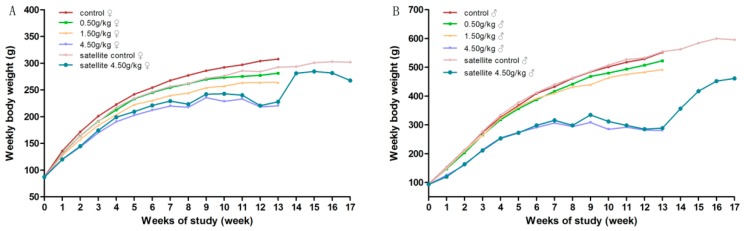
Average body weight of rats orally administrated with gardenia yellow for 90 days. (**A**) female; (**B**) male.

**Figure 3:**
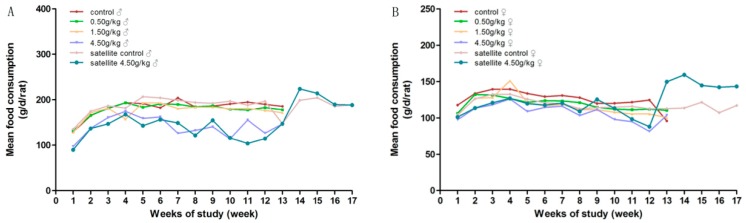
Average daily food consumption of rats orally administrated with gardenia yellow for 90 days. (**A**) female; (**B**) male.

During the recovery period, the clinical abnormalities, the decreased body weight and food intake of the high-dose satellite group were all significantly alleviated. Clinical symptoms, such as orange-colored urine and stools, were gradually returned to normal after three days discontinuation of administration and were considered to be of no clinical significance. The details are showed in Figure 2 and Figure 3.

#### 3.3.2. Urinalysis

No significant changes were found in the treated groups. Urinalysis parameters, including pH, GLU, PRO, BLD, SG, BIL, URO, KET and WBC were all within normal limits (data not shown).

#### 3.3.3. Hematological Parameters

As shown in Table 1, no adverse effect on the hematological parameters was found in the low-dose group as compared with the controls. While with the high-dose treatment, gardenia yellow was revealed to obviously affect the hematological parameters of rats. The influence on red blood cell parameters was shown as a dose-related decreasing trend of RBC and MCH, and an increasing trend of MCV, MCH and RET% in treated groups of both genders, with statistical significance in medium-dose and high-dose group versus the control group (*p* < 0.05). However, the values of the medium-dose animals still remained within the normal range.

In addition, the high-dose group also presented increases in WBC and NEUT% and decreases of LYMPH% of both genders (*p* < 0.05). The related changes in the medium-dose group were still within the normal range.

For the parameters on the blood coagulation system, the significant increase of PLT was shown in the high-dose group of both genders (*p* < 0.05). No significant dose-related change was revealed from PT or APTT (data not shown).

The changes of the hematological parameters in the high-dose group were observed to be alleviated to the normal range during the recovery period.

#### 3.3.4. Clinical Biochemical Parameters

As compared with the control rats, dose-related changes, including TP, ALB, ALP, BUN, CRE, GLU, CHOL and TG, were observed in the gardenia yellow treated rats, with statistical significance in the decreased TP, ALB, GLU, CHOL and TG, increased BUN and CRE of the high dose females, decreased TP and TG, increased CRE of the medium-dose females, decreased TP, GLU and increased CRE of the high-dose males, decreased GLU and increased CRE of the medium-dose males (*p* < 0.05). Among them, most changes of parameters of the medium-dose treated rats were still in the normal range and were considered to be not clinically significant. Moreover, the dose-dependent effect on the clinical biochemical parameters was more significant in the treated females than in the males.

The above changes were reversible during the recovery period, with no significance compared with the satellite control group (Table 2).

#### 3.3.5. Pathology

Gross pathology revealed the diffused coloration of organs like heart, liver, kidney, spleen and gastrointestinal tract of the treated groups, which were faintly discernible in the low-dose group and more obvious in the high-dose group. Treatment-related changes in the relative organ weights were evident in the high-dose group of both genders, with significance in the increased relative organ weights of spleen and the decreased relative organ weights of heart. Moreover, the females of the high-dose group were also observed with the significantly increased relative organ weights of liver and kidney.

Although a statistical decrease in the relative organ weight of thymus and a statistical increase in the relative organ weight of adrenals were observed in high-dose males (*p* < 0.05), no significance was found in the female treated groups versus the control. The statistical differences were considered to be incidental and of no clinical significance.

During the recovery period, all the pathology alterations had been moderated. The detailed data are presented in Table 3.

**Figure 4:**
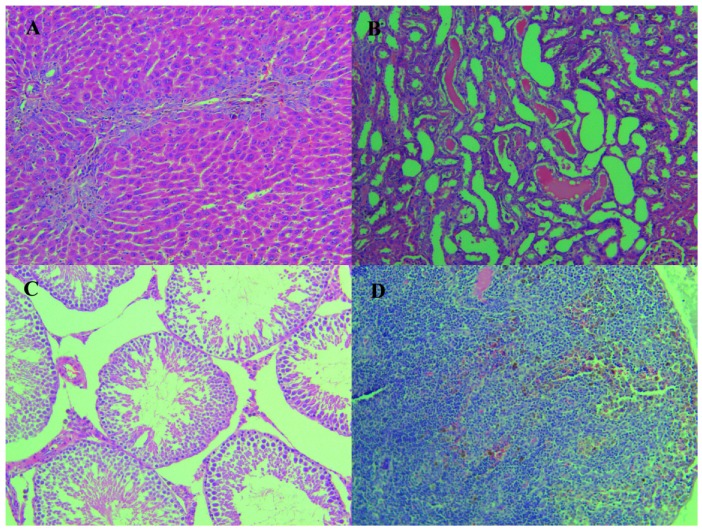
The presented images were the representatives of histopathologic changes of the live, kidney, lungs and lymphonodus in the high-dose group at the end of the 90 days administration. (**A**) (HE 10 × 20, hyperpigmentation), (**B**) (HE 10 × 20, Degeneration, necrosis, pigmentation of renal tubular epithelial cells), (**C**) (HE 10 × 20, Testicular interstitial pigmentation) and (**D**) (HE 10 × 20, Proliferation of macrophages and pigmentation in lymph nodes).

Upon histopathological examination, pigmentation was detected in livers, kidneys, lungs, testes and lymph nodes at the dose of 1.50 g/kg·bw/day or above, and more frequent and severe in the high dose group. In addition to the pigmentation, the high-dose group was also observed with oval cell proliferation in the liver, while the structure of hepatic lobule and portal area was still clear, and hepatocyte necrosis was not obvious. In the examination of kidneys, all the survived high-dose animals presented renal tubular epithelial cells, focal degeneration and necrosis while the medium dose animals did not (Table 4, Figure 4).

After 4 week withdrawal of the treatment, the coloration of liver, kidney and other organs in the high dose group remained dark and the incidence of pigmentation did not decrease yet. However, the damage in the kidney improved a lot with no visible tubular epithelial cells necrosis detected.

## 4. Discussion

With various properties, gardenia yellow has gained increasing popularity as a natural pigment. Aiming at providing the safety evaluating data, the present study was conducted to evaluate the acute and subchronic oral toxicity using S-D rat.

In the present acute oral toxicity study of gardenia yellow, no treat-related mortality occurred up to the maximal tolerable dose of 15.0 g/kg·bw. Based on the toxicity classification [17], gardenia yellow was assigned to the lowest toxicity class with an LD_50_ value greater than 5000 mg/kg and regarded as virtually non-toxic.

To investigate the target organ toxicity and determine the NOAEL of gardenia yellow, the experimental doses of the subchronic toxicity study were designed as 0.50, 1.50, 4.50 g/kg·bw/day, with the crocetin and derivative intake at the levels of 224.1, 672.3, 2016.9 mg/kg·bw/day, and geniposide at the 1.17, 3.51, 10.53 mg/kg·bw/day. According to the maximum use level of gardenia yellow in food as 1.5 g/kg from the Chinese National food safety standard for the use of food additives [6] and the assumed daily consumption level of solid food as 3 kg at physiological maximum, the maximum theoretical daily intake of gardenia yellow per person is calculated to be 4.5 g, or is equivalent to 0.075 g/kg·bw for an adult with average body weight of 60 kg. Moreover, the actual additive amount of gardenia yellow is related to its chromatic valence. The higher the chromatic valence, the lower the additive amount. Gardenia yellow E500 is a product with high chromatic valence, which cannot be directly added to food. Thus, the estimated human exposure of gardenia yellow E500 is highly conservative and much higher than the actual exposure.

After administration with gardenia yellow for 90 days, dose-related effects were observed on the body weight and food consumption level of gardenia yellow treated groups, which indicated the affected general health status of animals. Gardenia yellow at dose higher than 1.50 g/kg·bw/day was observed to interfere with gastrointestinal function with clinical symptoms, including watery and yellowed loose or liquid stools, which inhibited the normal intake of food and water and lead to reduced body weight, even severe malnutrition or dehydration. In accordance with our result, crocin from *Crocus sativus L.* (Saffron) was reported to induce diarrhea and weight loss in animals after intravenous administration at the dose of 180 mg/kg for 21 days [18].

In the present study, large-dose gardenia yellow was revealed with evident effect on the profile of hematological parameters. Animals in the high-dose group were observed with significantly decreased RBC, MCHC and increased MCV, MCH, RET%, which were probably related with the nutritional anemia due to gastrointestinal absorption disorder. In line with our results, previous studies showed the reduction of HB and HCT levels and total RBC count of rats and mice induced by saffron, with crocin as the main constituent [19]. In the safety evaluation of saffron (*crocus sativus*) tablets in healthy volunteers, saffron at higher dose (400 mg) was observed to slightly decrease some hematological parameters, such as RBCs, HGB, HCT and PLT [20]. Thus, crocin from gardenia yellow may also play an important role in the process. In addition, the animals from the high-dose group were observed with evidently increased WBC, NEUT% and PLT, as well as the decrease of LYMPH%, which was considered as the cellular response to infectious agents, tissue injury, or acute and chronic inflammatory process [21]. These changes are in line with the lesions in the liver and kidney revealed in the pathological examination.

The fluctuations of RBC and RET% in lower-dose groups did not exceed 20% of the control groups and were not considered to be clinically significant. Moreover, no significant changes were observed in the WBCs, NEUT%, LYMPH% and MONO% of the lower dose groups as compared with controls. In the previous studies with tested doses much lower than ours, no significant adverse effect on hematological parameters was reported in rats received gardenia yellow powder (as of genipiside at the dose of 56.3 mg/kg/day) for 13-week [22] or treated with crocin in feed at 3000 ppm (i.e., 143–166 mg/kg/day) [9].

With regard to the biochemistry indicators, the females produced a significantly decreased serum CHOL in the high-dose group (*p* < 0.05), which is below the normal range of our laboratory values and consistent with weight loss due to malnutrition. Meanwhile, the simultaneously decreased TG in the high-dose females (*p* < 0.05), although still within the normal range of laboratory values, was considered to be treatment-relevant. Moreover, crocin was reported to have the effect of lowering the levels of serum TG, CHOL and LDL-C by inhibiting pancreatic lipase in intestine [23,24]. Therefore, the decrease of CHOL and TG in the treated group maybe also related with the hypolimidemic effect of crocin. 

The dose-related decreased serum GLU levels in the treated groups of both genders were slight and still within the normal range in the laboratory, which was not considered of toxicological significance.

Significant differences were also shown in the decreases of TP in the high-dose group of both genders and the decrease of ALB in the high-dose females, with the TP value in the high dose males out of the normal range of our laboratory. TP and ALB may decline when protein is insufficient absorbed, reduced synthesized, or excessive lost, that mainly caused by gastrointestinal disorders, impaired hepatocellular function or kidney dysfunction. In our experiment, the above alterations are more likely to be linked with poor protein absorption, because the damage of hepatocytes is not serious inspected by the microscope and no proteinuria is found in urine examination.

No treatment-related changes were observed in the conventional liver toxicity markers such as ALT, AST, TBIL, except ALP. ALP is an enzyme mainly distributed in liver, bones, intestines and kidneys and commonly used for auxiliary diagnosis of liver and bone diseases. The elevated ALP in the high-dose females might be related to the liver damage which was further confirmed by the increased relative liver weight, intrahepatic pigment granules deposition and focal oval cell proliferation under microscope. When pigmentation was deposited in the liver, the weight of the liver increased. Although no obvious degeneration or swelling of hepatocytes was observed, the proliferation of oval cells derived from stem cells reflected the occurrence of the hepatic injury [25]. It has been reported that geniposide is metabolized to genipin (aglycone) through hydrolysis by b-glucosidase (intestinal bacteria), which is, in turn, cleaved to a dialdehyde derivative which reacts with polymers such as proteins, resulting in the induction of hepatotoxicty [10]. However, in Ding et al.’s study [13], geniposide at a normal dose of 24.3 mg/kg or less did not cause hepatotoxicity after 90 days repeated dosing, and the content of gardenoside in our high dose is only half of that, so the changes of liver are more likely to be attributed to the stimulation of the exogenous pigment granule deposition.

In addition, the significantly increased level of BUN in the high-dose females and the increased level of CREA in the medium-dose females and the high-dose groups of both genders were all beyond the normal range of the laboratory values and considered as good indicators of impaired renal function. Renal damage was further confirmed by the renal tubular epithelial cell degeneration and necrosis detected by microscope, where pigments were also present. The nephrotoxicity was demonstrated to be related to its inhibition of renal tubular cell transporters [14,26]. At the end of the recovery period, the changed renal function indexes decreased back to normal. Although the degeneration of renal tubular cells was still observed at various degrees in the satellite high-dose animals, no obvious necrosis was found. Therefore, the nephrotoxicity was considered to be reversible.

Histopathological examination also revealed pigmentation in the lungs and testes at the high-dose groups, except for the liver and kidney discussed above. Because there were no detectable lesions in the focal parenchymal cells, this deposition was not considered to be harmful.

## 5. Conclusions

Taken together, the LD_50_ of per oral gardenia yellow E500 to S-D rats was estimated to be more than 15.0 g/kg·bw and classified as virtually non-toxic. In the 90 days, oral subchronic test in S-D rats, the NOAEL of gardenia yellow E500 was suggested to be 0.50 g/kg·bw/day. When gardenia yellow E500 was gavaged at 1.50 g/kg·bw/day or more, pigmentation was present in some vital organs, causing local inflammatory reactions and tissue injury, such as gastrointestinal dysfunction, hepatotoxicity and nephrotoxicity, more notably in females.

According to the safety assessment for food additives of the WHO [27,28], the acceptable-daily-intake (ADI) is calculated by dividing NOAEL by safety factor, and the safety factor is generally determined as 100 (human being is 10 times more sensitive than animal and the difference range of sensitive within population is 10). Thus, in the present study, the ADI of gardenia yellow E500 was estimated to be 5 mg/kg·bw per day.

## Figures and Tables

**Table 1:** Hematology parameters of S-D rats orally administrated with gardenia yellow for 90 days.

Gender	Numbers of Animals	Dose (g/kg)	WBC (10^9^/L)	RBC (10^12^/L)	HGB (g/L)	HCT (%)	PLT (10^9^/L)	MCV (fL)	MCH (pg)	MCHC (g/dL)	NEUT%	LYMPH%	MONO%	EO%	BASO%	RET%
End of treatment period																
Female	10	0.00	4.7 ± 1.7	7.49 ± 0.38	140 ± 6	41.5 ± 1.6	855.5 ± 143.9	55.4 ± 1.9	18.7 ± 0.4	337 ± 5	30.2 ± 13.1	67.0 ± 13.4	1.1 ± 0.6	1.6 ± 0.7	0.0 ± 0.0	2.00 ± 0.53
	10	0.50	3.7 ± 1.4	7.20 ± 0.27 *	135 ± 4	40.9 ± 0.9	901.9 ± 115.7	56.9 ± 1.5	18.8 ± 0.4	330 ± 5 *	24.6 ± 5.0	72.6 ± 5.5	1.5 ± 0.5	1.2 ± 0.6	0.0 ± 0.0	2.24 ± 0.52
	10	1.50	3.6 ± 2.4	7.19 ± 0.23 *	138 ± 5	41.1 ± 1.6	889.0 ± 80.3	57.3 ± 1.5 *	19.0 ± 0.4	333 ± 8	24.1 ± 6.1	72.6 ± 6.7	1.5 ± 0.5	1.8 ± 1.1	0.0 ± 0.0	2.84 ± 0.57 *
	8	4.50	7.4 ± 1.8 *	6.42 ± 0.55 *	132 ± 10	41.0 ± 3.0	1027.4 ± 154.5 *	64.0 ± 2.7 **	20.6 ± 0.4 *	322 ± 9	39.1 ± 10.2	58.6 ± 10.3	1.0 ± 0.3	1.3 ± 0.8	0.0 ± 0.0	10.53 ± 2.10 *
Male	10	0.00	6.9 ± 2.4	8.55 ± 0.46	151 ± 7	43.5 ± 1.4	949.1 ± 100.8	50.9 ± 1.9	17.6 ± 0.5	346 ± 6	25.5 ± 4.4	71.7 ± 4.9	1.4 ± 0.7	1.5 ± 0.4	0.0 ± 0.0	2.08 ± 0.23
	10	0.50	6.5 ± 2.3	8.23 ± 0.19	148 ± 5	43.2 ± 1.5	943.8 ± 64.6	52.5 ± 1.6	18.0 ± 0.6	343 ± 3	27.9 ± 4.3	69.6 ± 4.4	1.4 ± 0.2	1.2 ± 0.6	0.0 ± 0.0	2.15 ± 0.25
	10	1.50	5.4 ± 1.0	8.04 ± 0.26 *	146 ± 4	42.5 ± 1.0	1019.3 ± 106.7	52.8 ± 1.9 *	18.2 ± 0.5	344 ± 5	25.7 ± 4.9	71.2 ± 4.6	1.6 ± 0.5	1.6 ± 0.7	0.0 ± 0.0	2.41 ± 0.28 *
	6	4.50	12.6 ± 2.9 *	6.67 ± 0.37 *	138 ± 10 *	42.1 ± 2.7	1230.8 ± 96.6 *	63.1 ± 2.2 **	20.6 ± 0.6	327 ± 6 *	46.8 ± 7.8 *	51.3 ± 7.1 *	1.0 ± 0.2	0.8 ± 0.6 *	0.0 ± 0.0	10.82 ± 1.54 *
End of recovery period																
Female	10	0.00	2.5 ± 0.7	7.21 ± 0.28	135 ± 5	39.4 ± 1.4	854 ± 76	54.8 ± 1.6	18.8 ± 0.4	343 ± 7	29.1 ± 5.9	68.3 ± 5.7	1.4 ± 0.8	1.2 ± 0.6	0.5 ± 0.2	2.23 ± 0.59
	9	4.50	3.4 ± 1.3	7.61 ± 0.30#	151 ± 7 ^#^	44.2 ± 1.9 ^#^	866 ± 64	58.0 ± 1.2 ^##^	19.8 ± 0.5 ^#^	342 ± 6	29.7 ± 5.6	64.8 ± 5.2	2.4 ± 0.9 ^#^	3.1 ± 1.0 ^#^	0.0 ± 0.0	1.14 ± 0.23 ^#^
Male	10	0.00	5.6 ± 1.7	8.61 ± 0.28	152 ± 5	43.6 ± 1.7	1027 ± 146	50.7 ± 1.3	17.6 ± 0.3	348 ± 4	28.1 ± 4.5	68.9 ± 5.1	1.6 ± 0.7	1.5 ± 0.4	0.0 ± 0.0	2.16 ± 0.21
	7	4.50	4.8 ± 1.1	8.39 ± 0.27	159 ± 4 ^#^	46.6 ± 1.0 ^#^	1051 ± 89 ^#^	55.5 ± 1.3 ^##^	19.0 ± 0.4 ^#^	342 ± 3 ^#^	28.8 ± 2.7	66.9 ± 3.1	1.9 ± 0.5	2.3 ± 1.2	0.0 ± 0.0	1.78 ± 0.29 ^#^

Each value represents mean ± standard deviation; * *p* < 0.05, ** *p* < 0.01, significantly different from control; ^#^ *p* < 0.05, ^##^ *p* < 0.01, significantly different from satellite control.

**Table 2:** Clinical biochemistry parameters of S-D rats orally administrated with gardenia yellow for 90 days.

Gender	Numbers of Animals	Dose (g/kg)	ALT (U/L)	AST (U/L)	TP (g/L)	ALB (g/L)	TBIL (μmol/L)	ALP (U/L)	GLU (mmol/L)	BUN (mmol/L)	CREA (μmol/L)	CHOL (mmol/L)	TG (mmol/L)
End of treatment period													
Female	10	0.00	32 ± 12	131 ± 38	64.4 ± 2.3	39.7 ± 1.8	0.68 ± 0.44	39 ± 12	7.14 ± 0.58	4.64 ± 0.49	58.7 ± 3.5	1.40 ± 0.25	0.71 ± 0.27
	10	0.50	27 ± 11	115 ± 18	63.2 ± 3.0	39.0 ± 2.0	0.40 ± 0.15	40 ± 14	6.84 ± 0.69	5.23 ± 0.60 *	65.5 ± 6.7 *	1.78 ± 0.27 *	0.43 ± 0.17 *
	10	1.50	25 ± 6	116 ± 26	60.9 ± 3.4 *	37.6 ± 2.6	0.51 ± 0.10	47 ± 22	6.75 ± 0.61	4.87 ± 0.74	72.6 ± 5.7 *	1.41 ± 0.22	0.35 ± 0.18 *
	8	4.50	26 ± 6	134 ± 18	55.5 ± 2.1 *	32.5 ± 1.0 *	0.74 ± 0.32	78 ± 29 *	5.69 ± 0.63 *	6.20 ± 0.77 *	83.1 ± 23.1 *	1.06 ± 0.37 *	0.11 ± 0.09 *
Male	10	0.00	27 ± 4	111 ± 17	53.4 ± 2.5	29.4 ± 1.2	0.55 ± 0.12	78 ± 15	7.72 ± 0.70	4.45 ± 0.45	47.2 ± 5.9	1.01 ± 0.30	0.85 ± 0.60
	10	0.50	25 ± 3	93 ± 4 *	52.9 ± 1.8	29.8 ± 1.3	0.61 ± 0.13	68 ± 17	7.40 ± 0.43	4.45 ± 0.35	49.2 ± 1.8	1.13 ± 0.27	0.66 ± 0.37
	10	1.50	26 ± 4	103 ± 18	54.2 ± 2.7	31.2 ± 1.0 *	0.85 ± 0.80	72 ± 17	6.73 ± 0.37 *	4.49 ± 0.49	54.1 ± 4.8 *	1.02 ± 0.21	0.41 ± 0.14 *
	6	4.50	36 ± 5 *	102 ± 14	48.5 ± 1.9 *	28.3 ± 0.9	0.88 ± 0.55	84 ± 12	5.49 ± 0.55 *	4.50 ± 1.46	69.9 ± 13.0 *	1.36 ± 0.29	0.50 ± 0.32
End of recovery period													
Female	10	0.00	30 ± 12	145 ± 28	69.2 ± 3.8	39.9 ± 2.7	1.8 ± 0.6	20 ± 5	6.92 ± 0.72	4.51 ± 0.34	67.0 ± 5.8	1.55 ± 0.38	0.46 ± 0.20
	9	4.50	25 ± 4	125 ± 26	63.2 ± 2.6 ^#^	34.4 ± 1.9 ^#^	0.9 ± 0.3 ^#^	33 ± 9 ^#^	6.50 ± 0.45	4.46 ± 0.68	61.7 ± 6.0	1.46 ± 0.22	0.46 ± 0.14
Male	10	0.00	35 ± 10	126 ± 18	60.3 ± 2.0	30.9 ± 1.3	1.2 ± 0.3	53 ± 24	8.27 ± 0.41	4.74 ± 0.44	58.9 ± 4.7	1.04 ± 0.27	0.67 ± 0.26
	7	4.50	37 ± 5	129 ± 20	57.9 ± 2.1 ^#^	29.8 ± 1.1	0.9 ± 0.1 ^#^	57 ± 14	7.06 ± 0.72 ^#^	5.61 ± 0.59 ^#^	60.6 ± 5.4	0.96 ± 0.35	0.31 ± 0.07 ^#^

Each value represents mean ± standard deviation; * *p* < 0.05, significantly different from control; ^#^ *p* < 0.05, significantly different from satellite control.

**Table 3:** Relative organ weights to brain (%) of S-D rats orally administrated with gardenia yellow for 90 days.

Gender	Numbers of Animals	Dose (g/kg)	Thymus	Heart	Liver	Spleen	Kidneys	Adrenals	Ovaries/Testes	Uterus/Epididymides
End of treatment period										
Female	10	0.00	0.21 ± 0.05	0.52 ± 0.04	3.99 ± 0.30	0.27 ± 0.04	0.88 ± 0.08	0.035 ± 0.012	0.27 ± 0.05	0.073 ± 0.012
	10	0.50	0.19 ± 0.03	0.51 ± 0.07	4.08 ± 0.47	0.28 ± 0.05	0.87 ± 0.08	0.037 ± 0.006	0.29 ± 0.08	0.071 ± 0.012
	10	1.50	0.18 ± 0.04	0.48 ± 0.06	4.19 ± 0.52	0.29 ± 0.05	0.91 ± 0.12	0.034 ± 0.008	0.27 ± 0.12	0.067 ± 0.010
	8	4.50	0.18 ± 0.07	0.46 ± 0.03 **	5.02 ± 0.54 **	0.43 ± 0.08 **	1.03 ± 0.09 **	0.034 ± 0.005	0.26 ± 0.04	0.090 ± 0.020 *
Male	10	0.00	0.26 ± 0.07	0.73 ± 0.09	6.30 ± 1.28	0.37 ± 0.07	1.50 ± 0.15	0.030 ± 0.006	1.56 ± 0.20	0.70 ± 0.10
	10	0.50	0.32 ± 0.13	0.81 ± 0.09 *	7.40 ± 1.25	0.41 ± 0.07	1.64 ± 0.20	0.038 ± 0.010 *	1.51 ± 0.21	0.69 ± 0.13
	10	1.50	0.22 ± 0.07	0.68 ± 0.05	6.43 ± 1.14	0.42 ± 0.04	1.48 ± 0.21	0.033 ± 0.007	1.65 ± 0.18	0.69 ± 0.07
	6	4.50	0.16 ± 0.07 *	0.59 ± 0.07 **	7.07 ± 1.17	0.55 ± 0.09 **	1.40 ± 0.34	0.041 ± 0.007 **	1.57 ± 0.09	0.65 ± 0.10
End of recovery period										
Female	10	0.00	0.16 ± 0.04	0.48 ± 0.16	3.80 ± 0.45	0.27 ± 0.04	0.93 ± 0.16	0.041 ± 0.007	0.31 ± 0.06	0.083 ± 0.013
	9	4.50	0.19 ± 0.04	0.54 ± 0.05	4.17 ± 0.40	0.33 ± 0.06 ^##^	1.02 ± 0.18	0.048 ± 0.007 ^##^	0.29 ± 0.04	0.090 ± 0.017
Male	10	0.00	0.27 ± 0.10	0.79 ± 0.07	6.89 ± 0.85	0.41 ± 0.06	1.61 ± 0.20	0.039 ± 0.010	1.75 ± 0.16	0.85 ± 0.08
	7	4.50	0.21 ± 0.07	0.72 ± 0.09	6.08 ± 0.80	0.42 ± 0.07	1.43 ± 0.17	0.037 ± 0.011	1.63 ± 0.10	0.83 ± 0.15

Each value represents mean ± standard deviation; * *p* < 0.05, ** *p* < 0.01, significantly different from control; ^##^ *p* < 0.01, significantly different from satellite control.

**Table 4:** Histopathological findings of S-D rats orally administered with gardenia yellow for 90 days.

Organ and Tissue Findings		End of Treatment Period (g/kg)								End of Recovery Period (g/kg)			
		0.00		0.50		1.50		4.50		1.50		4.50	
		♀	♂	♀	♂	♀	♂	♀	♂	♀	♂	♀	♂
Lungs	Pulmonary interstitial pigmentation, macrophage infiltration	0/10	0/10	-	-	-	-	1/8	1/6	0/10	0/10	1/9	0/7
Liver	Intrahepatic pigmentation	0/10	0/10	0/10	0/10	4/10	0/10	8/8	6/6	0/10	0/10	9/9	6/7
	Oval cell proliferation	0/10	0/10	0/10	0/10	0/10	0/10	3/8	3/6	0/10	0/10	3/9	0/7
Kidney	Tubular epithelial cells degeneration	0/10	0/10	0/10	0/10	0/10	0/10	8/8	6/6	0/10	0/10	9/9	7/7
	Tubular epithelial cells necrosis	0/10	0/10	0/10	0/10	0/10	0/10	8/8	6/6	0/10	0/10	0/9	0/7
	Tubular epithelium pigmentation	0/10	0/10	0/10	0/10	9/10	0/10	8/8	6/6	0/10	0/10	9/9	7/7
Testis	Leydig cell pigmentation	-	0/10	-	-	-	-	-	5/6	-	0/10	-	5/7
Lymph node	Lymphoid hyperplasia, pigmentation	0/10	0/10	0/10	0/10	0/10	0/10	3/8	5/6	0/10	0/10	1/9	3/7

Each value represents the number of animals with each finding/the number of the survived animals of the same sex.
